# Current approaches for modulation of the nanoscale interface in the regulation of cell behavior

**DOI:** 10.1016/j.nano.2017.03.020

**Published:** 2018-10

**Authors:** Hannah Donnelly, Matthew J Dalby, Manuel Salmeron-Sanchez, Paula E Sweeten

**Affiliations:** a Centre for Cell Engineering, Institute of Molecular, Cell and Systems Biology, College of Medical, Veterinary and Life Sciences, Joseph Black Building, University of Glasgow, Glasgow, Scotland, UK; b Biomedical Engineering Research Division, University of Glasgow, Glasgow, Scotland, UK

**Keywords:** Nanofabrication, Interface, Biomaterials, Extracellular matrix, ECM, Extracellular matrix, GFs, Growth Factors, PEA, Poly(ethyl acrylate), SAMs, Self-assembled monolayers, PEG, Poly(ethylene glycol), PCL, Poly(caprolactone), MSCs, Mesenchymal stem cells, FAK, Focal adhesion kinase, iPSCs, Induced pluripotent stem cells, ESCs, Embryonic stem cells, PMA, Poly(methyl acrylate), FN, Fibronectin, RGD, Arginine, gycine and aspartic acid tripeptide, PHSRN, Proline-histidine-serine-arginine-asparagine peptide sequence, EGF, Epidermal growth factor, EGFR, Epidermal growth factor receptor, VEGF, Vascular endothelial growth factor, PDGF, Platelet-derived GF, FGF, Fibroblast G, TGF-β, Transforming GF-β, BMP-2, Bone morphogenic protein-2, BMPR1a, Bone morphogenic protein receptor

## Abstract

Regulation of cell behavior in response to nanoscale features has been the focus of much research in recent years and the successful generation of nanoscale features capable of mimicking the natural nanoscale interface has been of great interest in the field of biomaterials research. In this review, we discuss relevant nanofabrication techniques and how they are combined with bioengineering applications to mimic the natural extracellular matrix (ECM) and create valuable nanoscale interfaces.

Cell behavior is regulated by soluble factors, cell–cell interactions and cell–extracellular matrix (ECM) interactions. Despite soluble chemical-based regulation of cell behavior traditionally being a topic of great interest, many researchers are now focusing on regulating cell behavior using physical cues associated with the nanoscale features of the ECM. It is well understood that cells respond to these nanoscale features and that they have the capacity to influence many cellular characteristics from morphology to gene expression (Figure 1). Consequently, modulation of the nanoscale interface using nanofabrication and use of nanoscale ECM features is a rapidly growing research area.^1^, ^2^, ^3^

**Figure 1:**
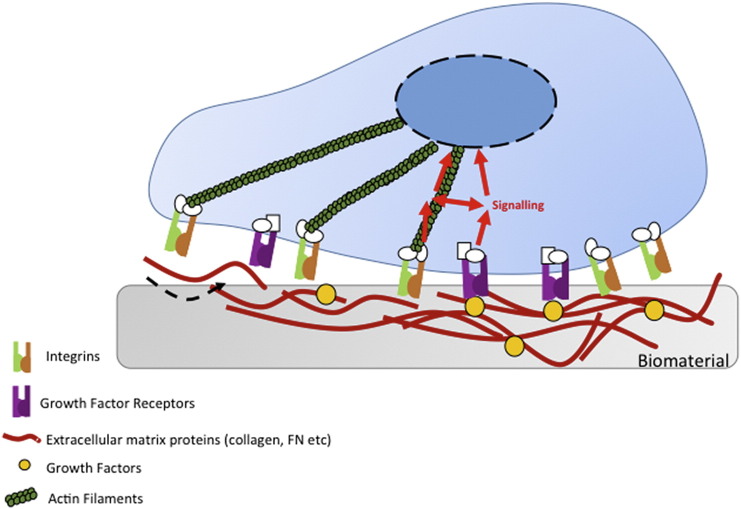
Integrin adhesion receptors link the extracellular matrix (ECM) to the actin cytoskeleton. The incorporation of ECM proteins and growth factors into biomaterials, allows for cells to respond to such materials *via* integrin binding. Following integrin-based binding of cells to the materials, signals can be transmitted to the nucleus *via* actin filaments.

As the interest in regulating nanoscale features and the nanoscale interfaces arising from these features has heightened, a range of nanofabrication techniques have been developed and used in the generation of physical cues capable of enhancing key characteristics of stem cells, such as their ability to differentiate down particular lineages. Techniques centered around lithography, electrospinning and molecular self-assembly have been shown to be effective in the production of nanoscale features on surfaces that can then be used as substrates for cell culture. What has been particularly striking about lithographic nanofabrication is that despite it primarily being a 2D process and thus cells are tested as 2D cultures, such surfaces are capable of generating similar levels of cell differentiation to that observed when cells are cultured in 3D ECM gels.^4^, ^5^ Although this initial discovery of the benefits of lithographic techniques is very promising, we are now seeing further advances in the use of lithography-based nanofabrication as it is being employed to spatially regulate cellular differentiation. Such spatial regulation of cellular differentiation is of prime importance as the progression in the research into nanoscale interfaces leads us out of *in vitro* testing and into *in vivo* testing and ultimately applications of these interfaces in the clinic.^6^, ^7^ Similarly, research into materials made *via* electrospinning has also shown that these interfaces are of potential clinical value, in that they have been shown to enhance bone formation without the problem of inflammation.^8^ Thus, it is becoming increasingly clear that manipulation of the nanoscale interface is of clinical significance.

Despite there being a broad appreciation of the diverse range of responses cells can make to physical nanoscale features, there is an increasing interest in using biomaterials to specifically mimic the nanoscale interface of the native microenvironment.^9^, ^10^ Cells respond to signals from the nanoscale environment associated with the ECM, including those coming from ECM proteins such as fibronectin, and also growth factors (GFs). This ability of cells to respond to ECM proteins has led to an increased interest in incorporating them into biomaterial research. This has consequently led to a new generation of biomaterials with nanoscale interfaces closer to those found in the ECM or, indeed, engineering ECM components as the nanoscale features.^11^, ^12^, ^13^, ^14^ This incorporation of ECM proteins has also opened up new avenues for research into cell behavior regulation in response to nanoscale interfaces, as not only do these ECM proteins have nanoscale features, there is also scope to engineer biological complexity through protein–protein interactions.^15^, ^16^ For example, it is now understood that fibronectin has a growth factor binding domain, which can be constitutively exposed when fibronectin is adsorbed onto certain polymers, *e.g.* poly (ethyl acrylate) (PEA), providing a means for tethering growth factors to substrates and therefore adding additional nanoscale features to those already existing due to the presence of fibronectin.^17^

This review will discuss current progress in understanding and exploiting cell–nanointerface interactions.

## Nanofabrication in the generation of nanoscale interfaces

### Lithography nanofabrication in the creation of nanoscale interface

Control of cell behavior at the nanoscale has led to development of suitable nanoscale interfaces and the development of techniques focused on creating nanoscale features and patterns (Table).^6^ The generation of surfaces featuring nanotopography relies primarily on the processes of lithography (pattern transfer).^18^

**Table:** Summary of materials used in nanofabrication techniques.

Technique	Materials	References
Electron Beam & Nanoimprint Lithography	1. Polycarbonate	1. Hart et al (2007)
	2. PMMA	2. Chou et al (1997); Biggs et al (2009)
	3. Polycaprolactone	3. Nie et al (2008)
	4. Polyethylene terephthalate	4. Antonini et al (2016)
	5. Poly(ethylene glycol)	5. Rundqvist et al (2006)
	6. Organosilane Self-Assembled Monolayers	6. Zhang et al (2005)
Photolithography	1. Poly(ethylene glycol)	1. Koh et al (2002)
	2. Poly-*N*-isopropylacrylamide	2. Albrecht et al (2005)
	3. Chitosan	3. Karp et al (2006)
	4. Biohybrid hydrogels (*e.g.* PEG-based hydrogels modified with peptide Arg-Gly-Asp (RGD)	4. Revzin et al (2003)
	5. Titanium dioxide	5. Qiu et al (2016)
X-ray Lithography	1. polyurethane	1. Diehl et al (2005)
	2. Poly (ethylene glycol)	2. Kim et al (2010)
Electrospinning	1. Polycaprolactone	1. Ganesh et al (2014)
	2. Chitosan	2. Lotfi et al (2016)
Molecular self-assembly	1. (RADS) motif polymers	1. Zhang et al (1999)
	2. Collagen	2. Aravamudhan et al (2016)
	3. Peptide-amphiphiles (PAs)	3. Yu et al (1998)
	4. Fibronectin	4. Llopis-Hernández et al (2016); Rico et al (2016)

One of the most high-resolution nanolitographical techniques is electron beam lithography, where an electron beam is focused on to substrates coated in electron sensitive resist. The resist can then be developed and etched to provide the lithographical step – commonly used now is reactive ion etch, or dry etch.^19^ It has been shown that it is possible to produce etches of 5-7 nm on surfaces, using an electron beam of 5 nm diameter.^20^ Electron beam lithography typically writes onto silicon wafers as it is a semiconductor industry driven technique. However, nanoimprint lithography can then be used to produce polymeric replicas by *e.g.* injection molding and these replicas have been used to understand cell response.^21^, ^22^, ^23^, ^24^ Further, electron beam lithography has also been used to successfully ablate precise patterns into self-assembled monolayers (SAMs).^25^ Highly regarded for its precision, electron beam lithography in conjunction with self-assembled monolayers is now being used for a range of cell studies, despite being a slow and costly method of fabrication.^26^, ^27^

Other types of lithography used for nanofabrication of biomaterials are X-ray lithography and photolithography, where soft X-rays of wavelengths between 0.4 and 5 nm or UV light is used as the radiation source.^19^, ^28^, ^29^ Soft X-rays and lithography have been used in the generation of biomimetic nanoscale interfaces with substratum features resembling the native basement membrane of the cornea, and has been used in studies focused on the use of biomaterials in corneal prosthesis development.^29^, ^30^, ^31^ X-ray lithography has also been used in the generation of nanoscale biohybrid gels composed of PEG-based hydrogels modified with peptide Arg-Gly-Asp (RGD) and also titanium dioxide.^6^, ^32^ In addition, X-ray lithography has been used to produce anisotropically nanofabricated substratum (ANFS) formed from scalable, biocompatible polyethylene glycol (PEG) hydrogel arrays, which have been used in the generation of macroscopic regions cardiac tissue constructs.^33^ The benefit of using X-ray lithography with the PEG hydrogel arrays is that it allows for the production of constructs that are structurally similar to the native myocardial environment, and that are also chemically similar, allowing maintenance of electrophysiological function.^34^

As touched on above, for biomaterials and tissue engineering research, polymers are of much greater interest than semiconductor-based materials such as silicon as they are biocompatible, flexible, processable and amenable to surface chemistry applications; in fact, chemical modifications of polymers are gaining increasing interest as a means of modulating the ECM and cell responses to it.^7^, ^11^ Thus, it is of significance that simply heating thermoplastic polymers to above glass transition temperature (Tg) and applying the pattern (in silicon or as a metalized shim for example) can produce high-fidelity (down to 5 nm) patterns in a wide range of polymers. We note that if the user is not concerned if the pattern is imprinted into polymer in the inverse, the original silicon master can be used as a stamp. However, sputtering and electroplating the master with nickel and then removing the master creates a negative against which polymer can be imprinted as per the originally defined pattern. Imprinting strategies from simply applying pressure by hand in low Tg polymers such as poly(caprolactone) (PCL) to injection molding for high throughput manufacture are now commonly used.^35^

Advances in nanofabrication over the last decade have meant that it has been possible to produce a diverse range of nanoscale materials able to be used in the generation of nanointerfaces for bioengineering.^36^ Many cell types have been used with these interfaces, but perhaps the best characterized are mesenchymal stem cells (MSCs). Due to their interaction with materials interfaces, MSC differentiation can be guided down different lineages, offering platforms for their regulation. Results showed that osteoprogenitors and MSCs cultured on disordered (but not random) topographies had raised levels of osteopontin and osteocalcin expression, suggesting that this particular material could be a candidate for bone-producing nanointerfaces. Subsequently, disordered nanotopographies have been produced into orthopedic-relevant materials, such as titanium, and shown to enhance bone formation *in vitro* and *in vivo.*^37^, ^38^

From this early work, production of biomaterials mimicking the nanoscale interface has been of great interest. For example, it could be noted that collagen type X, produced by cells during endochondral ossification and in large fracture repair, has a nanoscale pattern of controlled disorder – similar to the electron beam fabricated disordered surface features.^39^ In 2016, work was presented illustrating the use of photolithography to generate titanium dioxide nanotopographies, formed from nanorod arrays, that could be used in conjunction with smooth surfaces to spatially regulate osteogenic differentiation of MSCs.^6^ Such findings provide promise for the future of nanoscale biomaterial development, as they support the idea of production of materials with variation at the nanoscale interface, closely mimicking naturally-occurring nanoscale features that can recapitulate biological complexity *e.g.* at the bone/ligament interface. Further work in 2016 from Antonini et al has also shown the potential for the use of nanofabricated biomaterials to create biointerfaces. Also focused toward enhancing the osteogenic potential of MSCs, Antonini et al presented work using lithography-fabricated polyethylene terephthalate nanogratings, demonstrating their capacity to also enhance osteogenic differentiation of MSCs relative to flat controls.^40^

Nanoscale topography has been used to help elucidate cell-osteogenesis mechanisms. As cells adhere, they form large multi-protein complexes termed cell adhesions. Adhesions contain ECM adhesive strata containing integrins, a signaling strata containing *e.g.* focal adhesion kinase (FAK), a force transduction layer containing *e.g.* vinculin and an actin regulatory layer that localizes adhesions to the contractile microfilament cytoskeleton.^41^ Mechanical contraction is important as adhesions require both integrin anchorage to the ECM and force application in order to grow; and the longer adhesions become the more signaling that can be potentiated from the growth of the signaling strata. Upon integrin engagement, G-proteins are activated, including Rho, which drives actin/myosin sliding and hence cytoskeletal contraction. The growth of adhesions is critical for osteogenesis as osteoblasts are high-tension phenotypes that use super-mature adhesions (>5 μm long) to stabilize the contraction.^9^, ^42^ Nanoscale topographies that induce osteogenesis from MSCs tend to drive cell adhesion and it seems likely that induction of endogenous vitronectin production rather than fibronectin production is important (matrix proteins and integrins will be discussed in detail in the next section).^43^ Vitronectin better permits integrin bridging, adhesion and elongation allowing for breaks in the integrin strata through bridging in the force transduction layer.^44^

It is apparent that there is an ever-expanding future for the use of bioengineered nanoscale interfaces for the generation of biomaterials suitable for use in the clinic. For example, interesting results are being shown when stem cells including MSCs, induced pluripotent stem cells (iPSCs) and embryonic stem cells (ESCs) are cultured on nanoscale topographies. It has been observed that culturing ESCs on ridged nanotopographies induces increased cell alignment and elongation, but also reduces cell proliferation.^45^ Kingham et al showed that ESCs cultured on disordered nanotopograpies expressed MSC-like marker profile, and that it is possible to overcome challenges in regenerative medicine associated with the production of reproducible platforms for ESC differentiation.^46^ Further, work from Kong et al showed that it is possible to regulate gene expression of the Oct4 pluripotency gene in response to nanotopographical cues, which represents a milestone in human ESC research.^47^

### Nanofabrication techniques to recapitulate the ECM nanoscale interface

The ECM is an intricate meshwork of protein fibers such as those of collagen and elastin, ranging from 10 to 300 nm in diameter (Table).^48^, ^49^ The interwoven mesh is then also covered with nanoscale adhesive proteins including fibronectin, meaning that the ECM has a porous, nanoscale topography. In addition to these common features, the structure of bone comprises further hydroxyapatite crystals, highlighting the importance of the nanoscale interface in bioengineering. In an attempt to mimic the woven, fibrous nature of the ECM, biomaterials research employs techniques such as electrospinning and molecular self-assembly as methods of producing more ECM-like features compared to lithographical protocols (Figure 1).^27^, ^50^ Use of these techniques to generate nanostructured biomaterials is finding particular importance in MSC and orthopedic research.^51^

Electrospinning allows for the controlled deposition of polymer fibers on to a substrate, and is now a commonly used technique in the production of biomaterials. It is one of the most versatile methods for the generation of nanoscale fibers similar in morphology and dimension to the native ECM.^52^, ^53^, ^54^ For example, it has recently been reported that incorporation of nanohydroxapatite into PCL composite scaffolds has the ability to increase protein adsorption capacity of scaffolds, and also to enhance MSC proliferation and osteogenic differentiation. PCL is broadly used in such studies as it can be used to form composite scaffolds as it is biodegradable, has known mechanical properties and providing contrast capabilities that would allow the regenerative healing processes to be monitored *in vivo.*^53^

**Figure 2:**
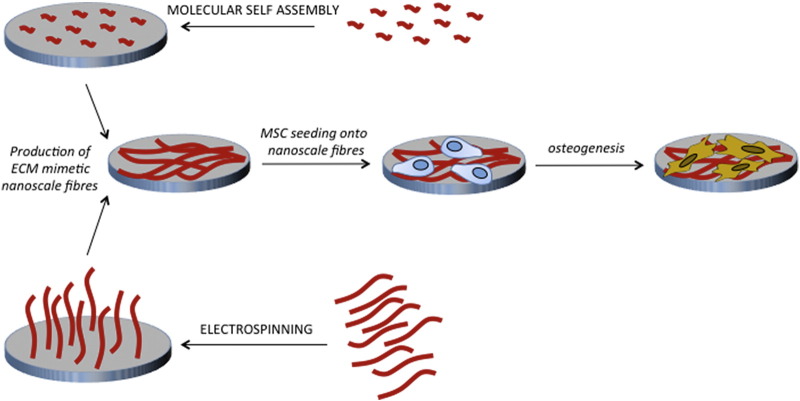
The use of molecular self-assembly and electrospinning in the generation of ECM-mimetic biomaterials. Molecular self-assembly involves the spontaneous self-assembly of protein structures and is often used in the generation of nanoscale ECM-mimetic biomaterials. Similarly, electrospinning is used to generate fibrous nanoscale structures, also capable of mimicking the nature of the natural ECM.

Work from Lotfi et al in 2017 has shown that chitosan-nanoelectrospun collagen membranes are also able to increase MSC proliferation and osteogenic differentiation compared to solid wall surfaces.^55^ This work elegantly demonstrates the careful pairing of the non-toxic, non-immunogenic, biocompatible, and biodegradable properties of chitosan with electrospinning. Use of this biomaterial was shown to increase bone formation *in vivo*, while causing no increased levels of inflammation (Figure 2).

Molecular self-assembly is a second type of nanofabrication gaining popularity as a result of it being naturally ubiquitous in biological systems, as well as its simplicity.^56^ This method for the synthesis of nanostructures is based around a spontaneous association of molecules under equilibrium conditions into structurally stable aggregates, and is used to fabricate novel, supramolecular architectures. Work on this method of nanofabrication has progressed considerably in the last three decades, and has shown that a variety of peptides can be designed to self-assemble into a monolayer on surfaces, allowing cells to adhere and respond appropriately.^57^, ^58^

Potential for the use of molecular self-assembly in generation of the nanoscale interface was highlighted in work by Zhang et al in 1999, where it was shown that self-assembly of oligopeptides containing cell adhesion motifs could be used to fabricate a range of surface patterns.^59^ These surface patterns could then be used to drive spatially regulated cell adhesion, a valuable feature in biomaterials where materials can be used as tools to help understanding of cell mechanisms. The function of these biomaterials is largely attributed to the presence of a C-terminal cysteine residue on the oligopeptide, which allows the peptide to covalently link to a bioinert gold-coated surface. Easy combination of a bioinert surface with oligopeptides capable of molecular self-assembly illustrates the vast potential for use of molecular-self-assembly in the generation of biocompatible nanoscale interfaces.

Further advances in molecular self-assembly have also shown that it is possible to use it for the production of small diameter nanofibers in the lowest end of the range of the natural ECM component collagen, a clear advantage over other nanofabrication techniques.^60^, ^61^ More recently, we have seen molecular self-assembly used similarly to aforementioned nanofabrication techniques, in that it is also being used in studies showing its capacity to regulate MSC fate with potential for use in orthopedics.^62^ Earlier this year, Aravamudhan et al published work showing that molecular self-assembly of collagen to form collagen nanofibers on a natural polymeric microporous structure could both enhance osteogenic differentiation of MSCs and enhance the biocompatibility of the biomaterial when subcutaneously implanted in mice.

Recently, an example of using materials as tools utilized supramolecular self-assembly of nanofibers in hydrogels of different stiffness.^63^ It has been shown that mimicking tissue stiffness drives stem cell differentiation.^2^ However, many hydrogels require crosslinking, coating and functionalization to change stiffness and allow cell interactions. New hydrogels have been designed from peptide nanofibers whereby stiffness was controlled using fiber density and no functionalizing or coating was required to permit cell adhesion. Such a clean system allowed for metabolomic comparison between MSC differentiation to express neural markers, chondrogenic markers and osteogenic markers at stiffnessess of 1, 15 and 32 kPa respectively. The lack of potential metabolite background from the nanofiber networks allowed identification of lineage specifying metabolites that, free from use of the gels, could be used to drive targeted differentiation.^64^ This demonstrates potential for new biomaterials in drug discovery.

Although collagen is a commonly researched protein in the area of molecular self-assembly, the study of other ECM proteins such as fibronectin (FN) and their self-assembly is also gaining attention. For example, studies have shown that FN can spontaneously form nanoscale networks in response to adsorption on PEA.^65^ In addition, data have been published showing FN nanoscale features induced as a response to poly(methyl acrylate) (PMA) and PEA, promote maintenance of MSC stemness, rather than lineage commitment. This work has been key in highlighting the future for the use of molecular self-assembly in the generation of nanoscale interfaces, which could be used in MSC niche design.

## Roles of peptides at the nanoscale interface of the extracellular matrix

The ECM is a dynamic network, supplying microenvironments with reservoirs of GFs and peptides, as well as integral structural support for organs, tissues and performing key roles in the stem cell niches. As well as looking toward topographical inspiration from the ECM, key signals from this micro/nanoenvironment are being incorporated into biomaterials; these include insoluble extracellular matrix macromolecules and diffusible or soluble molecules, such as GFs.^3^, ^66^

The ECM is extremely heterogeneous and tissue specific. However, there are three major classes of insoluble macromolecules: (1) structural proteins, such as collagen, laminin and elastin, (2) fibrous glycoproteins including fibronectin and vitronectin and (3) glycosaminoglycans (GAGs) such as keratin, hyaluronic acid and chondroitin sulfate, which are hydrophilic molecules with roles in buffering and cell adhesion.^67^ The secreted ECM exists as a network of these proteins acting as a nanoscale structure to support cells and provide an actively instructive interface to guide behavior as discussed above. Each of these proteins contains a plethora of binding, attachment and signaling capabilities that exist in numerous combinations of isoforms, ratios and topographical arrangements to produce the precise environment. In turn this leads to a wealth of molecular mechanisms that propagate this information into signals to cells to form complex tissues.

**Figure 3:**
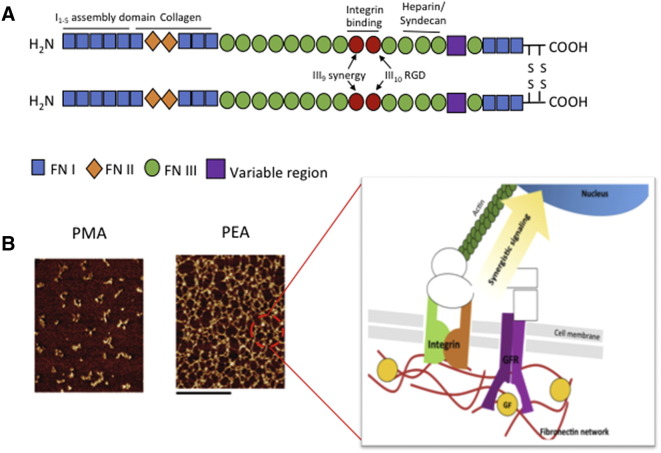
Integrins presented in synergy with growth factors. **(A)** Structure of fibronectin, showing location of major binding sites. The integrin binding region of FN is FNIII9-10, which lies adjacent to FNIII12-14, the growth factor binding region. **(B)** AFM images (phase magnitude) of FN adsorbed on material substrates. The scale bar is 0.5 μm. FN adsorption results in a self-assembled FN nanonetwork at the material interface of PEA but not PMA, this promotes synergystic signaling through integrins and growth factor receptors. (Reprinted (adapted) with permission from ^76^ Copyright 2015 American Chemical Society.)

The abundant ECM protein fibronectin (FN) is a ubiquitous dimeric glycoprotein with two subunits of ~250 kDa each, linked by intramolecular disulfide bonds. Each subunit contains three modular repeating units, type I, II and III, which mediate interactions with other FN molecules (FNI_1-5_ and FNIII_1-2_), other ECM components or integrins (FNIII_9-10_), and GFs (FNIII_12-14_) (Figure 3). Integrin α_5_β_1_ is well characterized as the primary receptor for FN; it contains an arg-gly-asp (RGD) site located in the type III_10_ repeat which has a three-dimensional presentation containing integrin binding pockets. Therefore, multiple integrins such as α_v_β_3_ also bind the RGD site, but they remain uninfluenced by the presence of an additional synergistic sequence from the FNIII_9_ repeat, Pro-His-Ser-Arg-Asn (PHSRN), that is required for full adhesive activity from the α_5_β_1_ receptor, enhancing the affinity of the integrins for the RGD loop over forty-fold.^68^, ^69^

Attempts to engineer ECM-derived adhesive peptides at the nanoscale interface to increase substrate bioactivity have focused on using recombinant fragments of the FN RGD domain adsorbed onto synthetic or natural non-fouling, non-absorbent materials. Many studies have demonstrated integrin-mediated effects on cell adhesion and migration as a result of RGD incorporation onto a range of materials such as alginate hydrogels, silk films and synthetic polymers.^70^, ^71^, ^72^ However, the bioactivity of these materials is significantly lower than when the full protein is used. Garcia's group has engineered recombinant FNIII_7-10_, a 39 KDa fragment of native FN modules 7-10 containing both the RGD and PHSRN synergy sites. The fragment maintains the native conformational spacing between the sites, as the structural orientation of these two domains is essential for synergistic effects, and increases in distance have been shown to lead to complete abrogation of α_5_β_1_ binding, cell spreading and signaling.^73^, ^74^ This FNIII_7-10_ fragment was immobilized to a support surface, non-adhesive albumin, at controlled ligand densities. Cells adhered to these functionalized substrates, spread and assembled focal adhesions containing α_5_β_1_, vinculin and talin, and this effect was abrogated upon blocking with integrin-specific antibodies where cell adhesion was completely eliminated.^74^, ^75^ The group also recently translated this fragment to coat stainless steel fixation screws in osteoporotic rats, implants coated with the FNIII_7-10_ enhanced bone-screw fixation by over 50% and bone-implant ingrowth by 30% after 3 months.^76^

Work from the Spatz group has also been particularly revealing, reporting that focal adhesion formation is dependent on the ability to ligate multiple RGD ligands. Using colloidal patterning and placing RGD groups on the resultant topographies has shown that RGD subunits need to be less than 70 nm apart for integrin gathering into mature focal adhesion to occur.^77^, ^78^, ^79^ The gathering of integrins allows formation of the signaling strata driving cytoskeletal tension. This allows the cells to manipulate the ECM, in effect gathering RGDs to facilitate further integrin gathering, growing the adhesion – potentially to form super mature adhesions capable of stabilizing the high degree of intracellular tension required for MSC osteogenesis.^43^

Native FN is a globular protein, and its unfolding leads to exposure of these cryptic binding sites for integrin and also GF binding, prompting interactions with other ECM proteins to form the fibrillar networks that the ECM is comprised of. The process of unfolding is usually initiated by integrins binding to FN dimers, which causes FN self-association through exposure of the N-terminal assembly domain, and this instigates contractile forces in the cell to expose more binding sites.^80^ However, it has recently been observed that certain polymeric materials can drive FN unfolding without cells.^11^

When testing polymer-based cell-free systems, it was observed that unfolding spontaneously arises when FN is adsorbed onto PEA surfaces, but not on control polymers (such as the closely related PMA^7^, ^11^, ^12^, ^81^). Simple physical adsorption of FN onto PEA leads to the molecular assembly of a physiological-like network of fibrils, exposing the cryptic binding sites of the FN molecule and this system has been shown to enhance biological activities, for example myogenic differentiation of murine C2C12 myoblasts (Figure 3).^82^

Another critical ECM protein is type I collagen, the most abundant protein in bone, where molecules form highly organized 3-dimensional scaffolds for cells. The primary collagen-binding integrin is α_2_β_1_ which recognizes the hexapeptide GFOGER motif.^83^ Collagen scaffolds are well-documented to induce osteogenesis of MSCs and matrix mineralization *in vitro*. However, recent efforts have focused on increasing the poor mechanical strength of collagen-based scaffolds for orthopedic applications.^84^, ^85^, ^86^ *In vivo* collagen fibers exist as a highly cross-linked network; by mimicking this process *in-situ* collagen cross-linking can be achieved, with one study subsequently mineralizing the scaffold by incorporating a composite of catecholamine's and calcium ions that form CaCO_3_, increasing the Young's modulus to near that of cancellous bone.^87^ However, we note that a caveat with many cross-linking strategies is that many agents used are cytotoxic. Collagen scaffolds, for these limitations, are now more frequently incorporated into multi-material implants as GF carriers.^88^, ^89^

## Incorporation of growth factors in the nanoscale interface

GFs are critical biological signals that regulate cell growth, stem cell differentiation and tissue healing. In developmental biology, GFs co-ordinate cell differentiation and tissue organization, showing their large potential in regenerative medicine.^90^ The ECM acts as a reservoir for GFs as most ECM proteins and GFs have reciprocal binding sites, allowing for GF sequestration.^91^ The localized ECM can therefore regulate GF signaling intensity and duration, and control spatial and temporal GF signaling. Many engineering strategies attempt to functionalize biomaterials to mimic this combinatorial ECM-GF-cell nanointerface inspired by native GF interactions.^92^, ^93^

Traditional applications of GFs in tissue engineering involve solubilization of the GF in the cell growth media of MSC cultures, but not only does this require supraphysiological concentrations of the GF, it also does not permit the spatiotemporal control of *in vivo* GF presentation.^94^, ^95^ Introducing GF carriers such as ECM GF-binding domains, enables tethering of the GF to the cell–material interface and thereby increases local bioavailability to cells. Further to this, GF receptors (GFRs) and integrins are able to form signaling complexes, the localization of integrin-binding and GF-binding domains on ECM proteins can therefore drive enhanced synergistic signaling to control cell behavior.^7^, ^15^, ^96^, ^97^ In simple terms, ECM-GF complexes provide highly targeted and amplified (through receptor co-localization) signals to the cells (Figure 1).

Approaches to functionalize GFs at the material–cell interface aim to control the release and spatial orientation of these GFs. However, emerging methods each pose their own individual drawbacks, with each GF also bringing its own unique properties for effective delivery. Upon covalent immobilization to a substrate, epidermal growth factor (EGF) was shown to increase cell activation compared to passive physical adsorption.^98^ Since this study many more have focused on covalently modifying material interfaces. Fan et al 2007, used a poly(methyl methacrylate)-graft-poly(ethylene oxide) (PMMA-g-PEO) comb polymer system to covalently tether EGF in controlled physiological-like concentrations. When compared to saturated GF concentrations they found a significant increase in cell survival and spreading. By controlling GF spacing they were able to either restrict or support EGF receptor (EGFR) homodimerization, while the covalent binding inhibited receptor internalization thereby potentiating sustained EGFR signaling.^96^ However, it must be considered that some GFs require internalization for function, or some, such as vascular endothelial GF (VEGF), act through a chemotactic gradient.^99^ The nature of covalent bonding of GF also has the potential to disrupt GF conformation or bind it such that it is not in physiological orientation.

Recently, it has been elucidated that the FN 12th and 14th type III repeat is a highly promiscuous GF binding domain; this domain has thus been the focus of approaches to tether GFs at the material interface. The FNIII_12-14_ repeat binds VEGF/platelet-derived GF (PDGF), fibroblast GF (FGF) and transforming GF-β (TGF-β) family GFs with high affinity.^13^ The close proximity of this GF binding-domain to the FNIII_9-10_ integrin-binding domain promotes co-localization of GFR and integrins, leading to increased cross talk and synergistic cell signaling.^7^ One study exploited this by generating a recombinant FN fragment containing the FN III_9-10_ and III_12-14_ repeats only, which were then covalently immobilized into a fibrin matrix. Here they showed stimulation with solutions containing PDGF and the FNIII_9-10_/FNIII_12-14_ fragment significantly enhanced GF-induced proliferation and migration of MSCs when compared to fragments containing only the FNIII _9-10_ or FNIII _12-14_ fragment, suggesting synergistic effects between the GF and FNIII_9-10_/FNIII_12-14_-mediated integrin signaling.^13^

Another recent study exploited the material-driven fibrillogenesis (molecular self-assembly) effects of the polymer PEA to provide physiological presentation of GFs.^7^ Spin-coated PEA surfaces were used to drive FN nanonetwork organization after physical adsorption of the protein. Network formation exposes the FN binding sites allowing tethering of the GF bone morphogenic protein-2 (BMP-2) to the FNIII_12-14_ domain, while simultaneously exposing FNIII_9-10_ to promote integrin engagement and co-signaling. Robust osteoblastic differentiation of MSCs was observed, molecular and genetic analysis alongside co-immunoprecipitation and of the BMP-2 receptor (BMPR1a) with integrin β1 confirmed osteogenesis was driven through enhanced canonical BMP-2 signaling as a consequence of a synergistic occupancy of GFRs and integrins. Upon implantation into a non-healing bone defect in mouse this material-based approach was able to drive full tissue regeneration.^7^

Compared to soluble administration of GFs, systems that engineer GFs to the material interface, including covalent protein binding, have been shown to be more effective in controlling cell fate and behavior at the nanoscale interface. However, these technologies do not exploit the synergistic effects of integrins and GFRs (Figure 3). Both Martino et al, 2011 and Llopis-Hernandez et al, 2016, target integrins and GFRs in synergy by exploiting native ECM components.^7^, ^13^

## Future perspectives

The current literature demonstrates that modulation of the nanoscale interface using nanofabrication and bioengineering approaches with GFs and proteins provides a promising means for further research and the development of physiologically mimetic biomaterials for use in the lab and in the clinic. It is apparent that the incorporation of synthetic ECM protein shapes, proteins themselves and engineering their GF binding and cell binding characteristics provide powerful tools for success in these future directions.

Further, mimicking GF presentation allows control of powerful GFs. We note that GFs are used widely in the clinic but that their use remains controversial and thus their impact limited. Typically, GFs are delivered at supraphysiological doses due to rapid initial propagation from the implant site, for example the current gold standard for *in vivo* bone repair is the INFUSE® bone graft which delivers BMP-2 absorbed into a collagen I sponge at a concentration of 1.5 mg/mL. Such high concentrations of GF are not only costly, but also lead to severe off-target effects, urging the FDA to release a public health notification of life threatening complications associated with this device.^100^ We have discussed material platforms that provide cell control by mimicking the native microenvironment through nanoscale topography, through engineered adhesion sites and through synergistic GF presentation. Translation of these novel technologies will provide better cell control and prevent the collateral damage being observed with the current uses of GFs in the clinic.^101^
